# Posterior cervical congenital dermal sinus tract: case report and review of literature

**DOI:** 10.1038/s41394-023-00575-5

**Published:** 2023-08-02

**Authors:** Amir Vokshoor, Harseerat Jajj, Tiffany Grunwald, Steven Kolker, Jack Petros

**Affiliations:** 1 Spine Surgery Section Chief, Providence Saint John’s Health Center, Pacific Spine Institute, Santa Monica, CA USA; 2 Institute of Neuro Innovation, Santa Monica, CA USA; 3 Department of Plastic & Reconstructive Surgery, Providence Saint John’s Health Center, Santa Monica, CA USA; 4 Department of Pathology, Providence Saint John’s Health Center, Santa Monica, CA USA

**Keywords:** Spinal cord diseases, Spinal cord, Pathology

## Abstract

**Background and importance:**

Congenital dermal sinus tract (DST) is a rare spinal dysraphism characterized by a persistent tract lined by epithelial cells, beginning at the epidermis and terminating in deeper tissue layers. With 1% of all congenital DST cases found in the cervical region, only 4% of all cases are diagnosed after the age of 20.

**Clinical presentation:**

In this case, a 65-year-old woman with a congenital DST at the cervical level presented with symptoms of neck and some arm pain, suboccipital headaches, and unique external characteristics. Neck Disability Index and visual analog scale were used to assess the patient’s preoperative and postoperative pain, and quality of life. Patient underwent an operative intervention, where the DST was surgically removed followed by interlaminar decompression at C1–C2, excision of the epidural component, and biopsy followed by plastic surgical repair. Pathology analysis indicated a squamous epithelial-lined sinus tract interacting with the dura. Most notably, a meningothelial proliferation with associated psammomatous calcifications was identified, similar to a meningioma.

**Conclusion:**

A review of literature was conducted to further discuss clinical and radiological presentation as well as to document the novel appearance of this congenital DST. As one of the oldest cases of DST, it demonstrated unusual pathological characteristics with a meningothelial proliferation, compatible with meningioma, reported at the epidural level.

## Background and importance

Congenital dermal sinus tract (DST) is a rare spinal dysraphism characterized by a persistent tract lined by epithelial cells, beginning at the epidermis and terminating in deeper tissue layers [1]. With an incidence rate of 1 case per 2500 live births, congenital DST is found in the cervical region in around 1% of all cases [2, 3]. In 2020, Petrov reported a clinical case of a 58-year-old as one of the oldest patients with congenital DST in the cervical region [2]. Adding to the current literature, we would like to report a 65-year-old woman with congenital DST at the C1–C2 level who presented with symptoms and pathological findings unusual for this entity.

## Clinical presentation

**Fig. 1:**
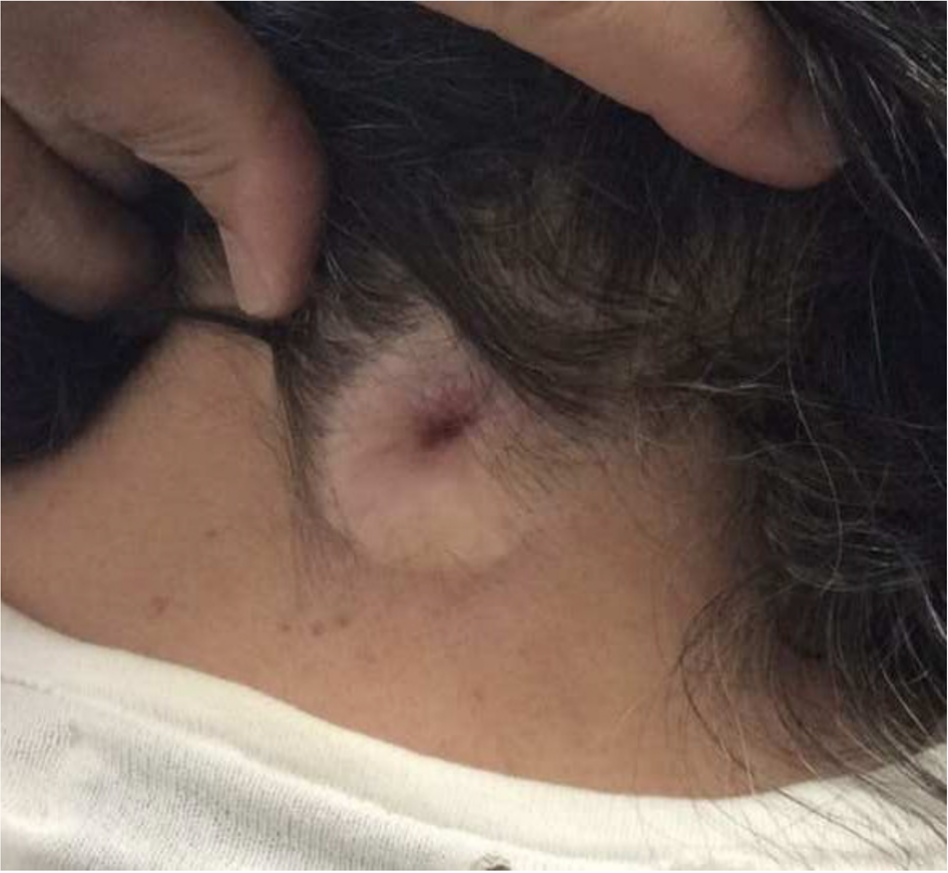
A preoperative image of the congenital DST in the posterior cervical region at the C2–C3 level. The DST presented as hypopigmented, no signs of infection, and contracted external sphincter-like outer boundary with hypotrichosis.

A 65-year-old female presented to the clinic with complaints of chronic suboccipital headaches consistent with occipital neuralgia. The patient’s chief complaints were a pain in the neck and head and tingling on the left side of the body, including the scalp, arm, leg, and lip. Patient has a family history of headache disorders, but no history of spinal problems. To alleviate the pain, the patient regularly took tramadol and even steroids. Patient mentioned that she was born with a “hole” in her neck, the persistent DST in the posterior cervical area, which gave her significant personal discomfort in its appearance as shown in Fig. 1.

**Fig. 2:**
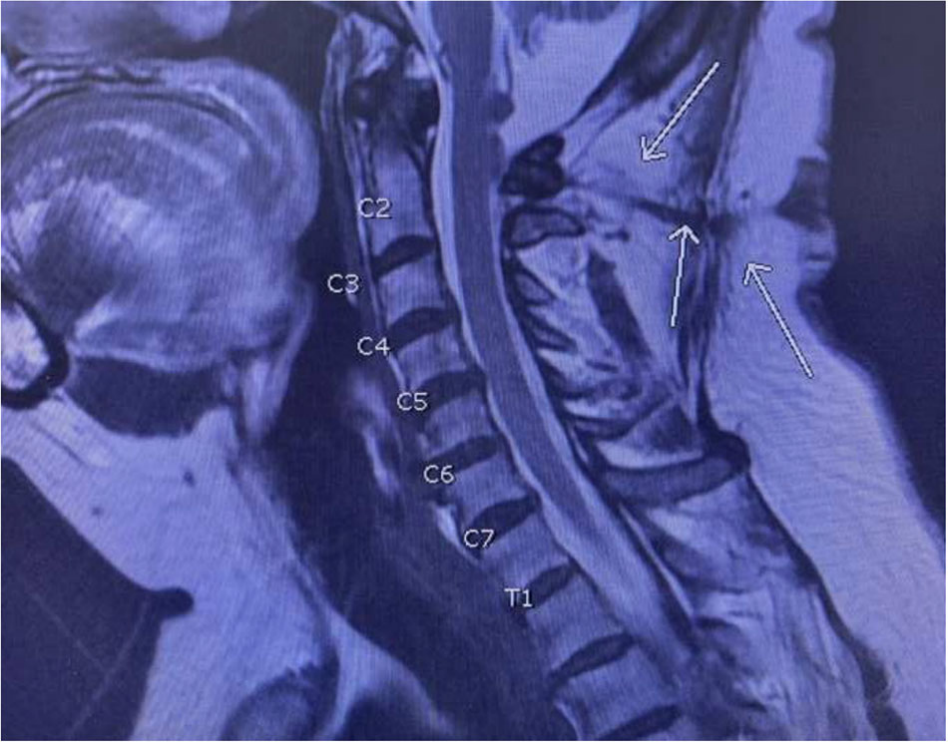
Preoperative cervical MRI indicated a tract from the epidermis at the C2 level, coursing rostrally and communicating with the dura at C1. The C1 bifid arch is traversed by the congenital dermal sinus tract.

Preoperatively, the patient reported a Neck Disability Index Score (NDI) of 46, and visual analog scale (VAS) neck 9.8. Neurological examination indicated the range of motion as stiff but relatively intact, motorically intact at 5/5, normal sensory, gait, and reflexes. The patient’s congenital DST was unique in its physical appearance as it was a hypopigmented, contracted external sphincter-like outer boundary with hypotrichosis, and no signs of infection. She denied any pain in that area or drainage from the opening. Contrary to a typical case of congenital DST, there was an absence of associated neurological dysfunction, swelling, infection in the area, or associated hemangioma. The cervical MRI shows C1 bifid arch traversed by the congenital DST as shown in Fig. 2. Initial impression indicates that the cervical osteoarthritis O–C1 on the left side is the likely cause of her neck pain and the suboccipital headaches could be associated with the DST, to be explored in the biopsy.

**Fig. 3:**
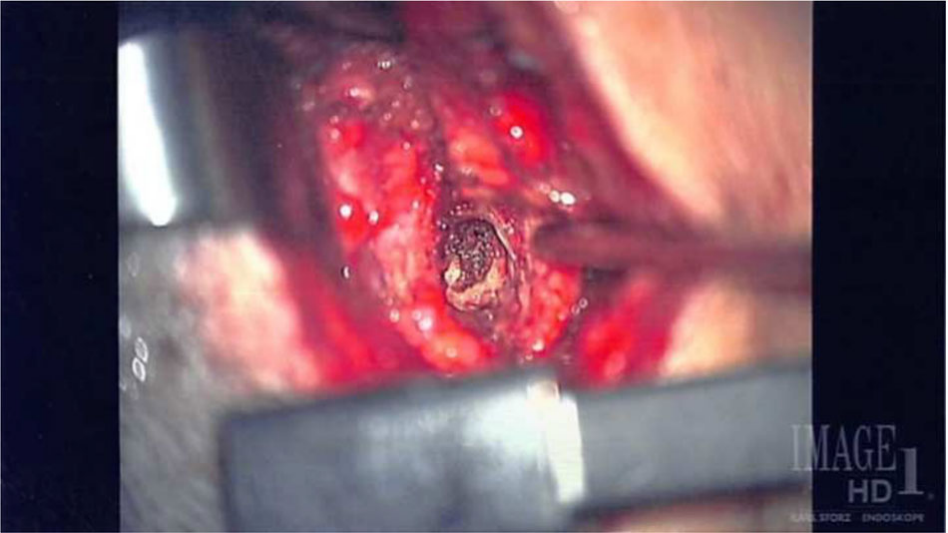
Intraoperative image of the DST communicating with the dura. Under high microscopic magnification the DST is visualized along its tract all the way to the epidural space and at the very center the C1 lamina is reached and the area of decompression is visualized.

**Fig. 4:**
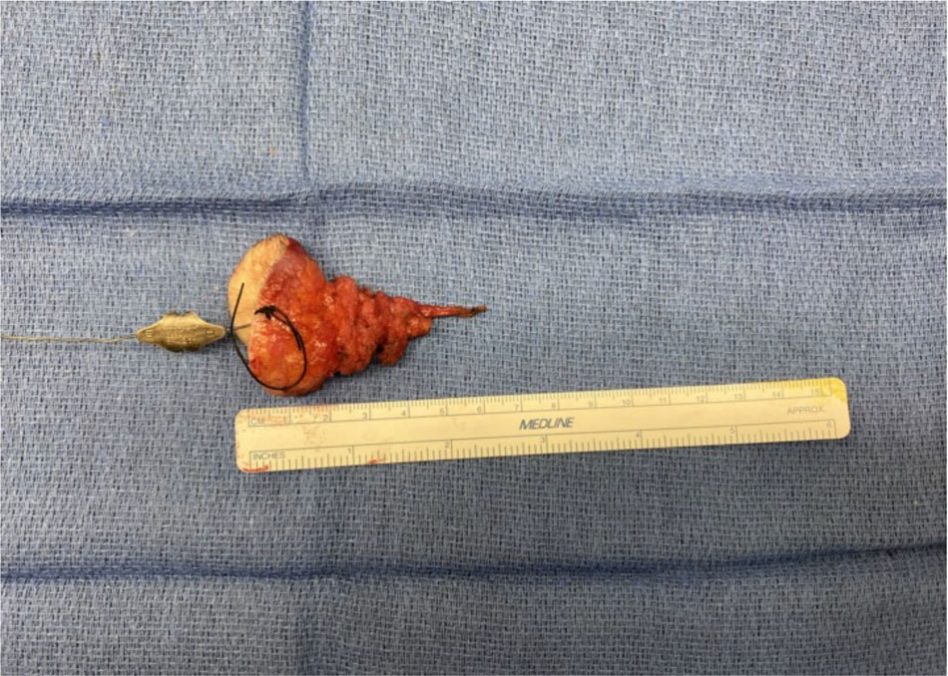
Intraoperative image of the sinus tract. Sinus tract length is measured to be 7.5 cm, including the redundant epidermis: the abnormality was submitted for pathological examination upon excision and this was a result of the gross total resection.

**Fig. 5:**
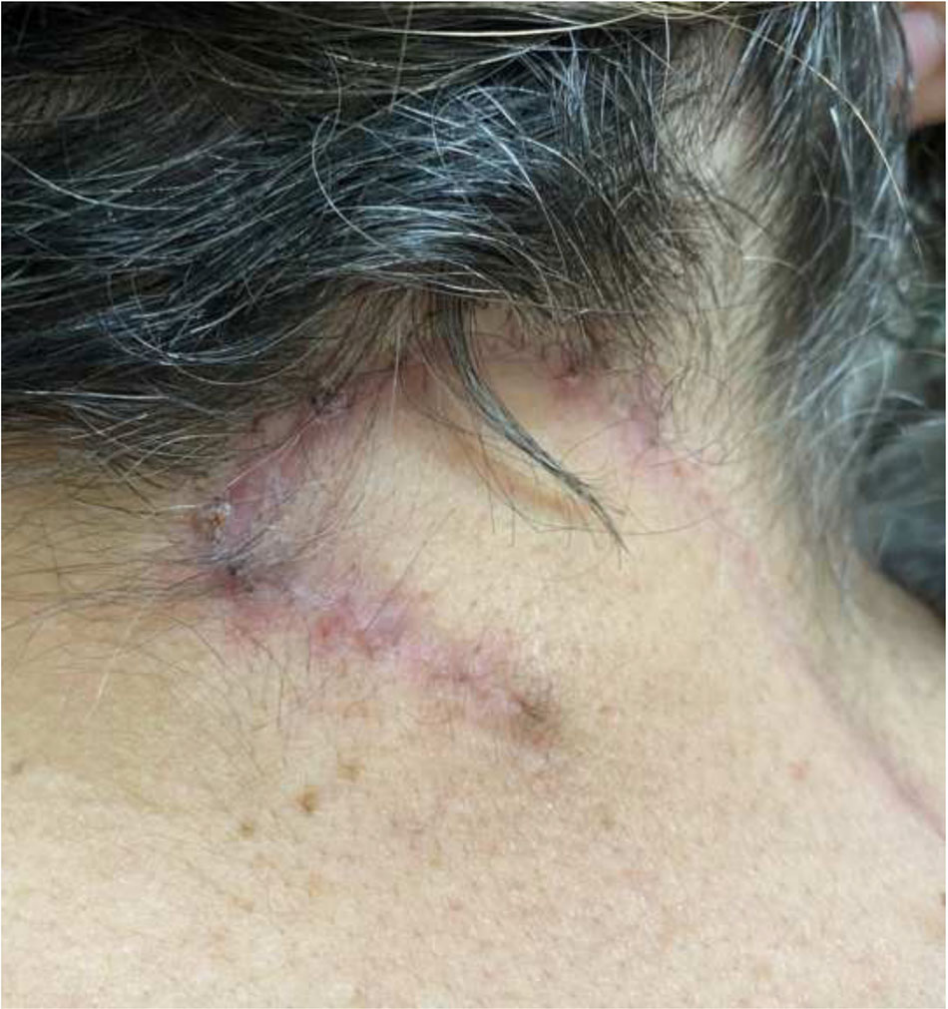
Closure of the congenital DST at the 3 months postoperative mark. Results demonstrated achieved by z-flap plastic surgical closure attained effective cosmesis.

The surgical intervention was an excision of the posterior cervical pathologic lesion that extends from the skin to the spine along with a plastic surgical repair. A circular posterior cervical incision around the DST was made, and the dissection was taken down along the tract past the cervicodorsal fascia. An excision of the congenital sinus tract and associated fistula tissues along with interlaminar decompression of C1 and top of C2 with epidural micro-dissection and excision was performed. The sinus tract was followed all the way to the C1 arch and the C1–C2 interlaminar space via a minimalistic coned-down dissection under microscopic magnification (Fig. 3). During the operation, the tract ended with a thin layer of dense tissue adherent to the dura mater. Subsequently, thinning of the C1 lamina and the top of C2 with a high-speed drill, revealed the terminal epidural tissue in the interlaminar space extending down from the bifid arch of C1. The specimen was removed off the dura mater after the laminotomy, and sent for pathologic analysis. There was no evidence of intradural extension or tethered cord. The true length of the sinus tract was measured to be 7.5 cm (Fig. 4). With the help of the plastic surgical team, a Z flap was rotated with fascial closure with vicryl in layers and primary closure was obtained with excellent cosmesis (Fig. 5). Pathological examination revealed an epithelial-lined sinus tract that extended into the epidural space with meningothelial proliferation and was interpreted as a classic (typical) meningioma.

**Fig. 6:**
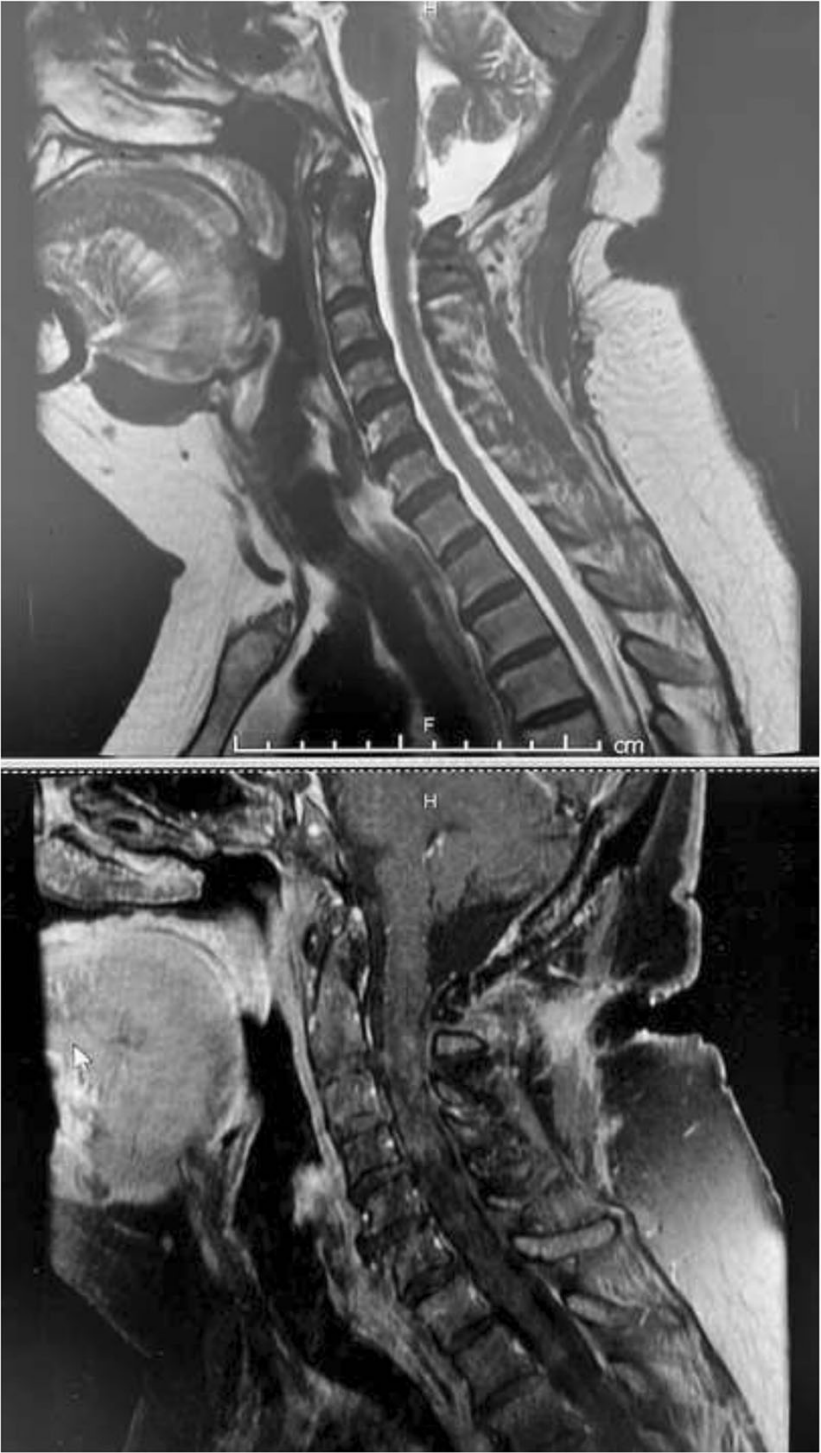
A MRI of the cervical spine where the top image shows the dorsal nodularity and the bottom contrast image shows no enhancement in the C1–C2 region. Indicating gross total resection.

At 3 months postoperatively, the patient was extremely satisfied and indicated resolution of her arm symptoms, C2 neuralgia, suboccipital headaches, and tingling on the left side of the body. The patient reported an NDI of 14 and VAS score of 0.1 (neck). Neurological exam remained stable. Five-month postoperative MRI indicates minimal dorsal nodularity without enhancement in Fig. 6.

## Discussion

Congenital DSTs are hypothesized to be the embryological result of an incomplete separation between the neural ectoderm and cutaneous surface ectoderm during the 3rd and 8th week of gestation [2]. Less than 1% of all congenital DST cases are found in the cervical region, where only 4% of the cases are diagnosed after the age of 20 [2]. The major clinical presentations and symptoms of congenital DST are hypertrichosis, abnormal pigmentation, infections, neurological deficits, skin dimples, erythema, hemangiomas, drainage of debris from the pit, tethered cords, inclusion tumors, and subcutaneous lipomas [2, 4–7]. Neurological deficits include motor weakness, sensory and reflex changes, and gait abnormalities [5, 8].

**Fig. 7:**
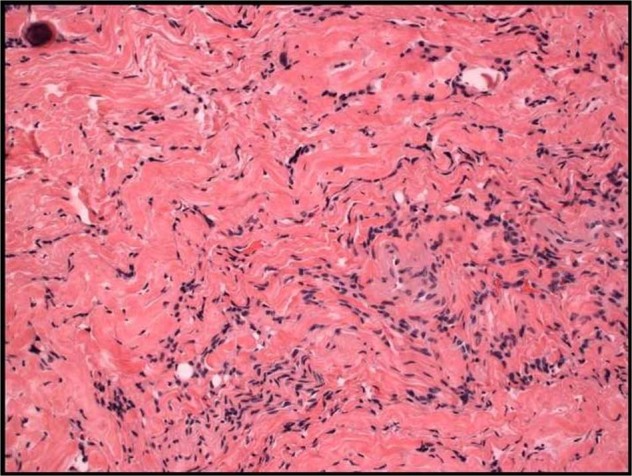
Pathology specimen photo micrograph under high powered 20× light microscope. Demonstrates Meningothelial cell proliferation embedded within a collagenous stroma with single psammomatous calcification in the upper left corner.

**Fig. 8:**
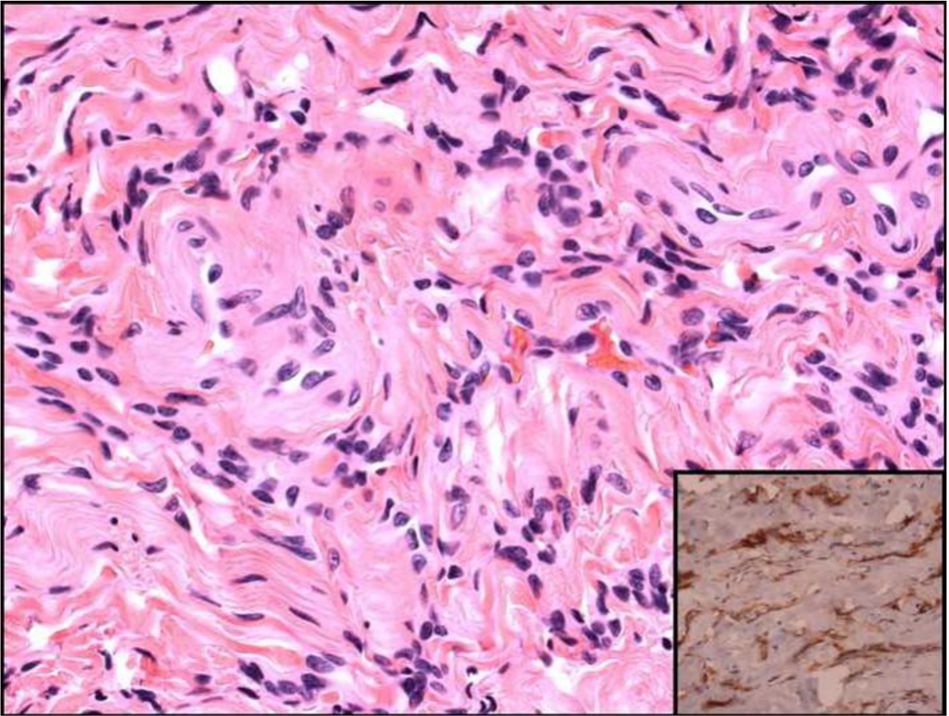
Pathology specimen photo micrograph under high powered 40× light microscope. High power image of meningothelial cell proliferation in a collagenous stroma, with immunohistochemical stain for SSTR2—a meningothelial marker—depicted in the lower right inset.

Our case demonstrates unique appearance, and pathology including squamous epithelial-lined sinus tract that extended from the epidermis, through the dermis, into epidural space. Meningothelial proliferation was found along with psammomatous calcifications (Figs. 7 and 8). The meningothelial proliferation was interpreted as a classic (typical) meningioma, fibroblastic type, WHO grade I. A meningothelial hamartoma was considered, but not favored as that entity is traditionally restricted to the dermis and subcutaneous tissue, often involves the scalp, and is not contiguous with the dura [9]. The area of meningioma was free of the cauterized dural margin, and completely excised. Contrary to the current literature on congenital DST, this patient presented as one of the oldest cases to be reported, uniquely with no previous infections and meningothelial proliferation. There was no intradural component or tethered cord [10].

## Conclusion

Diagnosed typically at birth or in early childhood, congenital DSTs are usually located in the lumbosacral region and rarely in the cervical region. Early diagnosis and treatment of congenital DST are important to prevent neurological deficits, infection and pain. To our knowledge, no previous reports of this type of DST with these unique external characteristics, spinal dysraphism with epidural meningioma, have ever been reported.

## Timeline


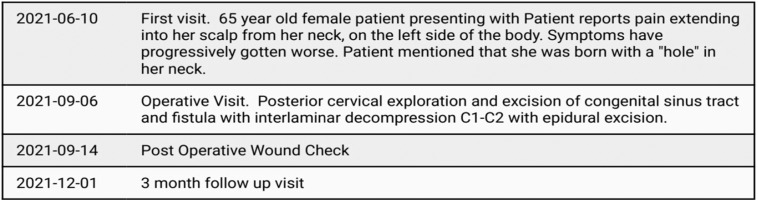


## Supplementary information

CARE Checklist

## Data Availability

The data that support the findings of this study are available from the corresponding author upon reasonable request.
